# The promotion of children's health and wellbeing: the contributions of England's charity sector

**DOI:** 10.1186/1756-0500-3-188

**Published:** 2010-07-13

**Authors:** Kamaldeep S Bhui, Lul A Admasachew, Albert Persaud

**Affiliations:** 1 Centre for Psychiatry, Barts and The London, School of Medicine and Dentistry, Old Anatomy Building, Charterhouse Square, London, UK; 2 Institute for Health Services Effectiveness, Work & Organisational Psychology Group, Aston University, Birmingham, UK; 3 The Centre for Applied Research and Evaluation International Foundation (careif), Barts and The London School of Medicine and Dentistry, Old Anatomy Building, Charterhouse Square, London, UK

## Abstract

**Background:**

Sports and arts based services for children have positive impacts on their mental and physical health. The charity sector provides such services, often set up in response to local communities expressing a need. The present study maps resilience promoting services provided by children's charities in England. Specifically, the prominence of sports and arts activities, and types of mental health provisions including telephone help-lines, are investigated.

**Findings:**

The study was a cross-sectional web-based survey of chief executives, senior mangers, directors and chairs of charities providing services for children under the age of 16. The aims, objectives and activities of participating children's charities and those providing mental health services were described overall. In total 167 chief executives, senior managers, directors and chairs of charities in England agreed to complete the survey. From our sample of charities, arts activities were the most frequently provided services (58/167, 35%), followed by counselling (55/167, 33%) and sports activities (36/167, 22%). Only 13% (22/167) of charities expected their work to contribute to the health legacy of the 2012 London Olympics. Telephone help lines were provided by 16% of the charities that promote mental health.

**Conclusions:**

Counselling and arts activities were relatively common. Sports activities were limited despite the evidence base that sport and physical activity are effective interventions for well-being and health gain. Few of the charities we surveyed expected a health legacy from the 2012 London Olympics.

## Background

Children's physical and mental health is adversely affected by social difficulties, low educational achievement, family mental illness, poor parenting, poverty and other traumatic life events [1-3]. Among the top 21 industrialised countries in the world, the UK ranks in the bottom third for children's health, with concerns about quality of their relationships with parents, risk taking behaviour, and relative poverty [4]. In many industrialised countries, emotional and behavioural problems are being identified as major causes of disability in children [5]. Consequently, the emphasis in social policy and public health is on preventing mental health problems, in part by strengthening resilience through health promoting services as well as building knowledge about healthy lifestyles. Be active, Be Healthy and Change4life are recent government initiatives that encourage better eating and physical activity [6,7]. Overall sport and physical activity are used at a public health level and as individual intervention [8-10].

Research indicates that getting involved in sport is an effective treatment for and an important part of preventive strategies for obesity, diabetes, heart disease, and other health conditions [11-14]. Arts and cultural projects also benefit the mental and physical health and well being of children [10-14]. It is well documented that the performance and production of art has long been valued in education, and encourages reflection of an artist's social, cultural and individual identity [15,16]. Sports have not been considered in the same light. However, it is anticipated that the London 2012 Olympic and Paralympic Games will provide a range of opportunities to support the Olympic ideals around the country and these benefits should not be restricted to London; indeed the Olympics promotes sports and values associated with sport throughout the world. These values include striving for excellence, doing one's personal best, fair play, improved inter-cultural understanding, social inclusion and encouraging positive views of disability [17]. The Olympics in London may be well placed to leave a public health legacy by inspiring children in the UK to be more active, have healthier lifestyles and take active roles in sustainable social and cultural interactions [18-20]. Studies of current levels of participation and encouragement for participation in sport and other activities related to healthy living and well-being are crucial. Such studies can provide baseline levels of activity, anticipating that if repeated following and during the Games, that there will be greater levels of participation and a measurable health legacy.

A number of sectors take responsibility for health and well-being. These actions are not restricted to public funded services, indeed communities are increasingly encouraged to take responsible actions to protect and preserve health. Statutory health services usually provide targeted interventions for specific health conditions with a shifting emphasis now to health promotion and prevention [21]. On the other hand, the charity sector (sometimes called the third sector or nongovernmental organisations) often addresses unmet need by providing services that include preventive and health promoting innovations, and these charities are usually are set up and shaped by local communities [22,23]. There is now a willingness to commission the charity sector so that it fills gaps in provision of statutory services [24]. Since the quality, capacity, and range of service provision in the charitable sector is not known, commissioning and service planning is not easy. The existing literature indicates very little research on the charitable sector's delivery of health promoting services for young people. This study maps provision for children by the charity sector focussing specifically on sports based and arts projects.

## Methods

### Sampling procedure

From a search of established charity websites (e.g. Guidestar UK and the Charity Commission) we confirmed the correct names and contact addresses for the official contacts of 500 charities that provide services for children under the age of 16. The charity commission's database has a record of all registered charities, and maintains public records about them. It also codes their aims and objectives on pre-determined categories against which their aims and objectives are classified and against which they are registered as charities by the charities commission.

We sent emails to each charity's official contact person and requested the name, telephone number and e-mail address of their chief executive, director, manager or chair. Following this, we undertook a web-based survey between April and August 2008 of chief executives, directors, managers or chairs. A first e-mail asked participants to access and complete an on-line survey; after a three month period a second reminder was sent to charities not responding to the first round. A second non-response was followed by telephone follow up and interview.

### The web-based survey

Initially, we reviewed the aims and objectives of charities as set out on the Charity Commission's database. These aims and objectives clustered into 20 categories used in the Charity Commission and Guidestar UK websites. For each of the 20 categories of aims and objectives, we asked a stem question, followed by additional questions about specific activities which are considered key areas of provision by National Children's Bureau, the Every Child Matters Agenda; the Sure Start Scotland mapping and evaluation exercise and Children's Services Mapping (CSM). Thus, endorsing any of the 20 stem question triggered additional detailed questions about activities of relevance to that particular aim and objective. Overall there were 133 additional questions. For example, for charities promoting mental health we asked about the provision of mental health promoting telephone help-lines. We collated these responses to estimate how many charities overall provide sports activities and arts activities (Table 1). All stem questions had an open response box for activities not listed. We also asked if charities were involved in generating a health legacy for the 2012 London Olympics. A zero response to any question indicated not endorsing a specific listed activity and also not mentioning it in an open response box.

**Table 1:** The aims and objectives of charities and their service provisions

**Aims and objectives of charities**	Percent* % **(n/N)**	**Provision of sports ** **services %** **(n/N)**	**Provision of arts & cultural** **Services %** **(n/N)**
Tackle educational exclusion	39.4 (65/165)	1.5 (1/65)	10.8 (7/65)
Tackle crime and anti-social behavior	31.5 (53/165)	32.1 (17/53)	50.9 (27/53)
Promote religious tolerance	12.7 (21/165)	0	4.8 (1/21)
Promote the well being of Black and Ethnic Minority	24.2 (40/165)	37.5 (15/40)	52.5 (21/40)
Promote the well being of asylum seeker and refugees	16.4 (27/165)	22.2 (6/27)	25.9 (7/27)
Promote gender and sexual equality	19.4 (32/165)		
Tackle unemployment	15.2 (25/165)		
Promote the well being of children and young people with special need	38.8 (64/165)		39.7 (25/63)
Tackle poverty	15.8 (26/165)	26.9 (7/26)	
Promote mental health	39.4 (67/165)	19.4 (13/67)	32.8 (22/67)
Promote sexual and general good health	22.4 (37/165)	32.4 (12/37)	
Promote the well being of children/young people who experience parental strain	33.3 (55/165)	14.5 (8/55)	30.9 (17/55)
Support young carers	13.3 (22/165)	14.3 (3/22)	38.1 (8/22)
Support looked after children	15.8 (26/165)	20.0 (5/26)	44.0 (11/26)
Support young run aways	7.3 (12/165)	16.7 (2/12)	44.0 (11/26)
Support gypsy and transient children	6.1 (10/165)	10 (1/10)	40 (4/10)
Support those who experience homelessness	13.9 (23/165)	21.7 (5/23)	39.1 (9/23)
Support substance misusing children/young people	13.3 (22/165)	31.8 (7/22)	36.4 (8/22)
Support children who have become sex workers	6.1 (10/165)	10 (1/10)	40 (4/10)
Provision of services for rural and isolated	7.9 (13/165)	15.4 (2/13)	30.8 (4/13)

^† ^Since a charity can contribute to more than one row, the percentages do not add up to 100%.

In our sample, sporting activities were provided by 36 charities (22%) while arts and cultural activities were provided by 59 charities (35%). Only 13% of the respondents indicated that they were involved in any form of health legacy for the London Olympics to be held in 2012.

The web tool and survey questionnaire were piloted on five people on five different occasions. Using information from the pilot, they were altered to improve the attractiveness of the web tool, the survey content validity, and the clarity of questions. For example, a list of ethnic group categories was incorporated in the survey rather than asking an open ended question. The piloting involved reviewing answers in open boxes and ensuring that these could be coded to one of the twenty categories of aims and objectives wherever possible, minimising reliance on the open response boxes. Descriptive statistics are presented (N and %). A favourable ethical opinion was given by Queen Mary research ethics committee.

## Results

In total, we received complete information from 167 charities (N = 500, 33.4%) which agreed to complete the survey; this represents a third of the sample charities that provide services for children under 16 in England. This response rate was not unreasonable for a web-based survey [25]. All non-responders were followed up by telephone. Information was collected by telephone from just over a third of 167 charities (N = 62, 37%). Charities that required telephone follow ups differed from those that did not, by annual reported expenditures of under £1,000 or over £1,000,000 a year. Of the 333 charities that did not take part in the survey, the majority (83%) were unable to provide information in a telephone follow up only because the relevant named person was not available at the time of the follow up phone call. Six percent of charities requested the original web-based survey to be redirected to a different person, 4 percent were too busy at the time of the survey and 3 percent did not feel the profile of their charity was reflected in the survey questions. The great majority of charities in our sample (59%) are based in London.

### The service provisions of charities

Table 1 shows the aims and objectives of the 167 charities that took part in the survey using the categories used by the charities commission. The table shows the prominence of sports or arts activities. The charities in our sample were most likely to tackle educational exclusion and promote mental health (39%). Over a third (38.8%) promote the well-being of children and young people with special needs. Disability, learning difficulty and other physical and mental impediments that hinder children from functioning were incorporated in the definition of special needs. Other aims and objectives range from those that tackle crime and anti-social behaviour to charities promoting the physical and mental health of children.

### Mental health promoting charities

Table 2 demonstrates the types of activities reported by charities that promote mental health. Face-to-face information and advice for service users were the most frequently provided activities. Only 11 charities (16%) provide telephone help lines, 19% promote sporting activities and 32% deliver art and cultural activities.

**Table 2:** Type of services provided in order to promote the mental health of children

**Type of Services**	**Frequency**	Percent*
Provision of advice through telephone helpline	11	16.4
Provision of face-to-face information and advice	38	57.6
Provide advice and information for teachers	26	39.4
Provide advice and information for parents	35	53.0
Provide counseling for children and young people	41	63.1
Provide training about mental health	24	36.9
Providing information to young people about the dangers of drug use and its impact on their mental health	14	21.5
Promote art and culture	22	32.8
Promote sport	13	19.4
Promote involvement in environmental activities	5	7.7
Campaigning in schools and other relevant places	10	15.4
Provide education into the prevention bullying and other abuse	28	43.1
Other, please specify	16	24.6

^† ^Since a charity can contribute to more than one row, the percentages do not add up to 100%.

## Discussion

The findings show a significant emphasis on counselling services and arts projects, but less on sport. Despite the existing evidence base on the benefits of sports activities, only a fifth of the overall sample offered this service.

Children's involvement in the 2012 London Olympics is supposed to be significant because they will take part as participants, spectators and organisers. Sir Steve Redgrave, regarded as one of the greatest Olympians, was appointed as the government's 2012 Olympic champions to inspire young people's participation. It is expected that children's levels of activity and involvement in sport will increase with demonstrable health benefits. Future surveys of young people in East London and nationally should show improvements in self-reported resilience and wellbeing. This legacy should ideally be demonstrable for all cultural groups, if the Olympic vision of transforming the nation's attitude to sport and intercultural communications is to be met. The charity sector could significantly contribute to local promotion of sport and the health legacy, but commissioners need to engage with the charity sector more actively. This survey provides useful baseline data but needs to be supplemented by additional surveys of young people. A future surveys are essential to measure and map the health legacy of the 2012 London Olympics. For example, the impacts may emerge from regeneration, new housing and better transport, new leisure facilities, as well as greater participation in sport. The different effects will have to be disentangled.

The limitations of this study should be mentioned. Our survey may have been too selective, with less participation by charities providing more sports, or other social interventions. However, we did not recruit by reference to any particular form of charity other than those providing services for children. We could not demonstrate any differences in the published aims and objectives of charities that participated in this study and those that chose not to take part. As the survey originated in a psychiatry research group, albeit an environmental and cultural psychiatry research group, it may have selectively attracted charities that provide mental health services. Potential research participants might have assumed that we were interested only in mental health providers. However, this is not reflected in the reasons for non-participation, with only 4% suggesting the survey did not reflect their charity's interests.

## Conclusions

In summary, sport and arts projects benefit mental and physical health; however, sport may not be prioritised by service providers, service users, and the public themselves as an intervention for well being in children. This may explain why few charities anticipated that they would contribute to a health legacy of the 2012 London Olympics.

## Competing interests

The authors declare that they have no competing interests.

## Authors' contributions

KSB was the grant holder and PI. KSB, LAA and AP performed the literature review. LAA undertook the web-based survey and data analysis. All authors read and approved the final manuscript.
